# Preparation and electroactive phase adjustment of Ag-doped poly(vinylidene fluoride) (PVDF) films†

**DOI:** 10.1039/c9ra08763j

**Published:** 2019-12-04

**Authors:** Seung-Hyun Kim, So-Jeong Park, Chang-Yeol Cho, Hong Suk Kang, Eun-Ho Sohn, In Jun Park, Jong-Wook Ha, Sang Goo Lee

**Affiliations:** Interface Materials and Chemical Engineering Research Center, Korea Research Institute of Chemical Technology Daejeon 34114 Republic of Korea sgoo@krict.re.kr; School of Chemical Engineering, Sungkyunkwan University Suwon 16419 Republic of Korea

## Abstract

The crystallinities of Ag-doped poly(vinylidene fluoride) (PVDF) films were modified by removing Ag^+^ using a novel washing process, which allowed control of the ratio of γ- and β-phases. The polarity of the composite film without Ag^+^ removal through the washing process reached 98%, and the β-phase content in the total electroactive phase was increased to 61%, according to Fourier-transform infrared spectroscopy. When Ag^+^ were removed through a process involving several cycles of washing, filtering, drying, and re-dissolving, the highest ratio of the γ-phase was increased to 67%, 28% higher than that before washing. This showed that Ag^+^ induced β-phase formation while Ag nanoparticles induced γ-phase formation, and that the ratio of γ- and β-phases in PVDF composite films can be controlled to suit specific applications by this washing process.

## Abstract 2

The ratio of γ- and β-phases of Ag-doped poly(vinylidene fluoride) films was controlled by removing Ag^+^ using a washing process.

## Graphical abstract

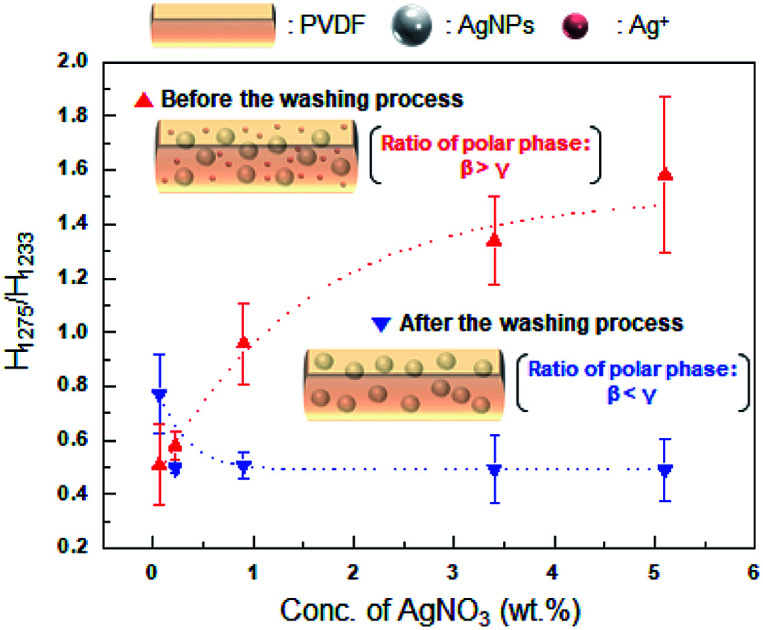

## Introduction

Extensive research has been conducted over the last few years on the development of high-performance energy harvesters for various applications, including self-powered sensors, smart skins, wearable electronics and portable electronics.^1–3^ Inorganic materials with high piezoelectric coefficients, such as BaTiO_3_, PbZr_*x*_Ti_1−*x*_O_3_ (PZT), ZnO, ZnSnO_3_, and GaN, have attracted attention because they possess nanostructures that facilitate electricity harvesting from mechanical energy.^4–12^ However, the rigidity, brittleness, processing difficulties, and toxicity of these materials have limited their possible applications.^13–17^

Polymer-based materials, on the other hand, are lightweight, flexible, and easy to process. Poly(vinylidene fluoride) (PVDF) is attractive because of its excellent pyro-, piezo-, and ferroelectricities.^18–21^ Moreover, flexibility, light weightness, easy processing, and presence of five crystallite phases (α, β, γ, δ, and ε)^22–25^ impart excellent mechanical properties, high thermal and chemical stabilities, and biocompatibility to PVDF.^26–29^ The polar β- and γ-forms are facilitate electrical applications.

Among the crystalizes polar phases with piezoelectric effect can be easily formed by external treatment such as stretching, heat treatment and poling (applying a high electric field) and crystalline induction of the film due to mixing with filler such as nanoparticles have.^26,30–32^ However, the crystallization induction of the film through an external treatment method has a disadvantage that it can induce an undesirable structural transformation and an incomplete crystal phase.^33,34^ Conversely, nanocomposite film formation of nano-fillers and PVDF can meet the requirements of device fabrication because it not only promotes the formation of polar phases in PVDF, but also improves the mechanical properties. Typically, different types of inorganic fillers are doped in PVDF to induce electroactive phases and improve electrical and mechanical properties.

Current methods for inducing crystalline polar phases with the piezoelectric effect in PVDF include stretching, heat treatment, poling, and addition of nanoparticle (NP) fillers to the PVDF matrix.^26,30–32^ While most of these methods can induce undesirable structural transformations and incomplete phases, the addition of NPs to PVDF promotes polar phase formation and improves mechanical properties.^33,34^ AgNPs have been widely studied as additives because of their high electrical and thermal conductivity, excellent catalytic activity in oxidation and reduction systems, effective anti-bacterial/anti-fouling action, and relatively low cost.^35–38^ However, the piezoelectric characteristics of AgNP/PVDF composite films depend on their electroactive phase composition, which is difficult to control using current manufacturing methods.

The aim of this study was to change the crystallinity of Ag^+^/AgNP/PVDF composite films by removing Ag^+^ through a new washing process and consequently control the ratio of γ- and β-phases. Compared to the afore-mentioned physical methods for modifying crystallinity, washing has not been well-studied or standardized. Herein, a washing process is outlined that allows for the removal of Ag^+^ from Ag^+^/AgNP/PVDF composite films to increase the ratio of the γ-phase.

## Results and discussion

**Fig. 1:**
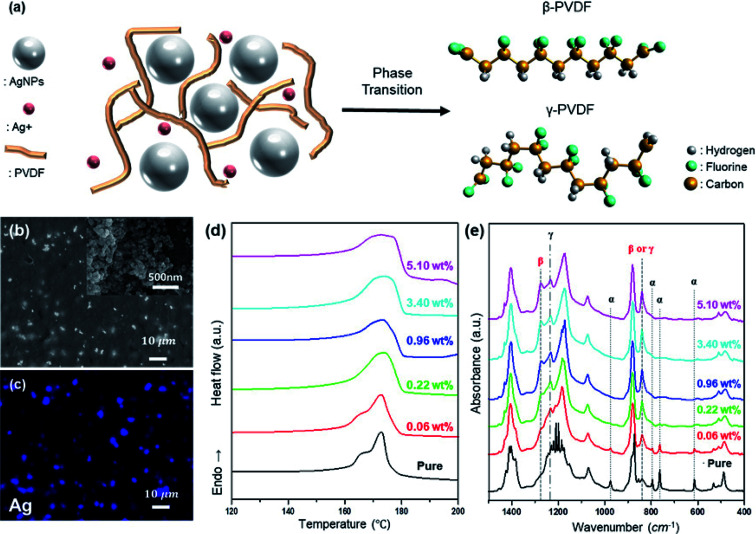
Preparation and analysis of Ag^+^/AgNP/PVDF composite film. (a) Schematic of Ag^+^/AgNP/PVDF composite film preparation. (b) SEM images (Insets show the SEM images of AgNPs), (c) EDS element mapping images of Ag atoms, (d) DSC melting endotherms, and (e) FT-IR spectra of Ag^+^/AgNP/PVDF composite films.

Studies on electroactive phase formation in PVDF nanocomposite films are mainly performed through FTIR spectroscopy, in which electroactive phases yield peaks at 840 cm^−1^.^39^ In addition, β-phase induction using AgNPs was reportedly effective in inducing electroactive phase formation in PVDF.^40^ However, when PVDF and AgNO_3_ are mixed for the formation of the PVDF/AgNP composite, and the electroactive phase is induced through film production, unreacted Ag^+^ remains, as shown in Fig. 1(a). The remaining Ag^+^ reacts with PVDF separately from AgNPs, thus affecting the electroactive phase induction of PVDF. To confirm this, the properties of the prepared Ag^+^/AgNP/PVDF films were studied. Ag^+^ and AgNPs are uniformly distributed in the prepared Ag^+^/AgNP/PVDF film, as shown in Fig. 1(b and c). The electroactive phase of PVDF is formed by PVDF–AgNP interaction; as the amount of Ag is increased, the reaction between PVDF and Ag is increased. Therefore, the electroactive phase content and melting temperature *T*_m_ are increased with increasing Ag, as shown in Fig. 1(d). In addition, the AgNPs and Ag^+^ induce electroactive phase formation in PVDF. As shown in Fig. 1(e), pure PVDF films prepared at 10 wt% and 80 °C show peaks at 975, 795, 764, and 614 cm^−1^ due to the α-phase. As the amount of Ag precursor is increased, these α-phase peaks disappear because of AgNP–PVDF and Ag^+^–PVDF interactions; instead, peaks at 840, 1233, and 1275 cm^−1^ from the polar β-and γ-phases appear. PVDF also has different melting points depending on the phase in which it exists.^41^ Pure PVDF has α- and γ-phases, and for these phases the peaks appear around 167 °C and 174 °C (Fig. 1(d)). Upon increasing the amount of Ag, the α-phase disappears and the γ-and β-phases emerge. The peaks appearing at 165 °C and 174 °C correspond to the γ- and β-phases. AgNPs and Ag^+^ tend to induce γ- and β-phase formation respectively. The peak corresponding to the β-phase appears with a polar solvent from hydrogen bonds between the PVDF chains and the F bridging AgNO_3_ and the PVDF chain.^42^ The γ-phase, meanwhile, is induced when the surface charges of AgNPs cause vibrational motion through electrostatic coupling between PVDF dipoles. Some parts of the PVDF polymer experience a polar induction from the reaction between the negative charge on the AgNP surface and the δ^+^, generated around the H atom of PVDF. However, the degree of polarity of the affected parts does not affect the rest of the polymer chain, leading to a γ-phase in the form of T_3_GT_3_G'.^43^

As the amount of Ag precursor (AgNO_3_) increases, so does the quantity of unreacted Ag^+^. Therefore, in comparing Ag 0.96, 3.40, and 5.10%, the total polar phases are similar, but as the amount of precursor increases, the peak at 1275 cm^−1^ representing the β-phase is heightened. Increasing the amount of hydrazine fraction of Ag^+^ reduction is also increased and thus the amount of AgNPs. As a result, the β-phase is decreased and the γ-phase is increased as shown in Fig. S1.†

**Fig. 2:**
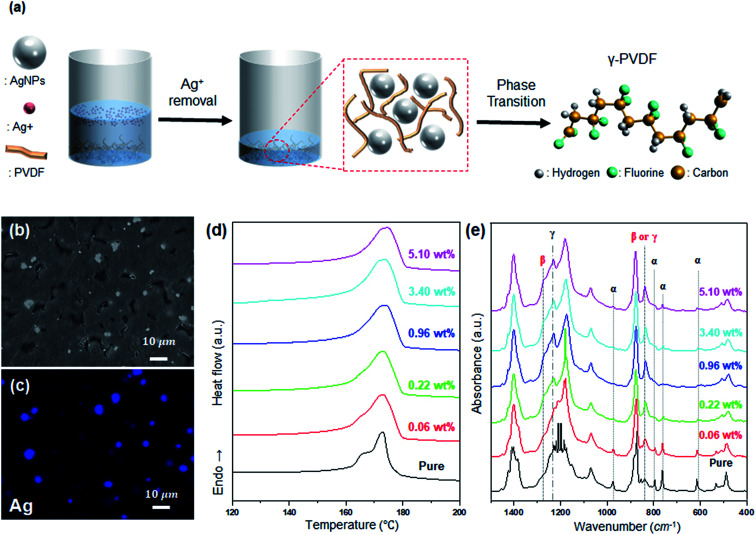
Preparation and analysis of AgNP/PVDF composite film. (a) Schematic of AgNP/PVDF composite film preparation. (b) SEM images, (c) EDS element mapping images of Ag atoms, (d) DSC melting endotherms and (e) FT-IR spectra of AgNP/PVDF composite films.

The modification of the PVDF electroactive phase to γ-phase is achieved through washing, as shown in Fig. 2(a). As shown in Fig. 2(b and c), the AgNP/PVDF composite films are formed such that AgNPs are uniformly distributed in the PVDF. These films lack the Ag^+^–PVDF interactions described above. Therefore, as AgNP concentration is increased, the electroactive phase induction of PVDF by AgNPs is increased, and the electroactive phase is also increased only up to a certain concentration. However, since excess AgNPs interfere with the reaction between the adjacent AgNPs and PVDF, the total percentage of the polar phase is decreased.^44,45^ As a result, the film *T*_m_ increases until 0.96 wt%, as shown in Fig. 2(d), but decreases again starting from 3.4 wt%. As shown in Fig. 2(b), α-phase is predominant until 0.06 wt%, but after 0.22 wt%, γ-phase is generated and α-phase is decreased. Therefore, the *T*_m_ of the film is increased, and is highest at 0.96 wt% with the highest γ-phase. *T*_m_ decreases from 3.4 wt% because of the predominance of the α-phase.

Unlike Ag^+^, AgNPs induce γ-phase formation in PVDF. As shown in Fig. 1(e), when the Ag concentration is increased, the peaks at 975, 795, 764, and 614 cm^−1^ from the α-phase are diminished, and peaks at 1233 and 1275 cm^−1^ appear from the polar phase. However, as shown in Fig. 2(e), when Ag^+^ is removed and only AgNPs are present, peaks at 975, 795, 764, and 614 cm^−1^ from the α-phase are decreased with increasing Ag concentration, and the peak at 1235 cm^−1^ from the γ-phase is increased. These results demonstrate that Ag^+^ induces β-phase formation and that of the polar phase composition of PVDF can be controlled.

Data analysis using XRD was also performed to confirm the control over the polar phase ratio of PVDF. As shown in Fig. S2(a and b),† the Ag/PVDF composite film before the washing process had a peak of 18.5° corresponding to the γ (202) plane and broad peaks at 20.2° and 20.5° corresponding to the β (110) and (110/200) planes. However, after washing, only peaks at 18.5° and 20.2° appeared, as shown in Fig. S2(c and d).† The peak at 38.5° indicated the presence of AgNPs.

The crystallinity (*X*_c_) in the Ag/PVDF composite film can be quantitatively calculated using the following equation,^46^1
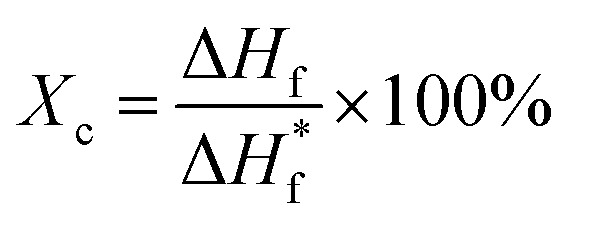
where 
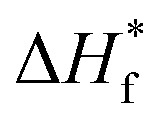
 and Δ*H*_f_ are the enthalpy of melting for 100% crystalline PVDF (104.7 J g^−1^) and the Ag/PVDF composite film, respectively. The crystallinity of the pure PVDF film is 41.67%, while the crystallinity of Ag/PVDF composite films slightly increases (refer Table S1†). After washing the films, the AgNP/PVDF film showed the highest crystallinity of 42.71% at 5.10 wt% whereas the Ag^+^/AgNP/PVDF film with both γ- and β-phases before washing also showed a maximum crystallinity of 43.33% at 5.10 wt%. This indicates that not only the γ- and β-phases of the film increased, but also the total crystallinity slightly increased.

Data analysis using XRD was also performed to confirm the control over the polar phase ratio of PVDF. As shown in Fig. S2(a and b),† the Ag/PVDF composite film before the washing process had a peak of 18.5° corresponding to the γ (202) plane and broad peaks at 20.2° and 20.5° corresponding to the β (110) and (110/200) planes. However, after washing, only peaks at 18.5° and 20.2° appeared, as shown in Fig. S2(c and d).† The peak at 38.5° indicated the presence of AgNPs.

**Fig. 3:**
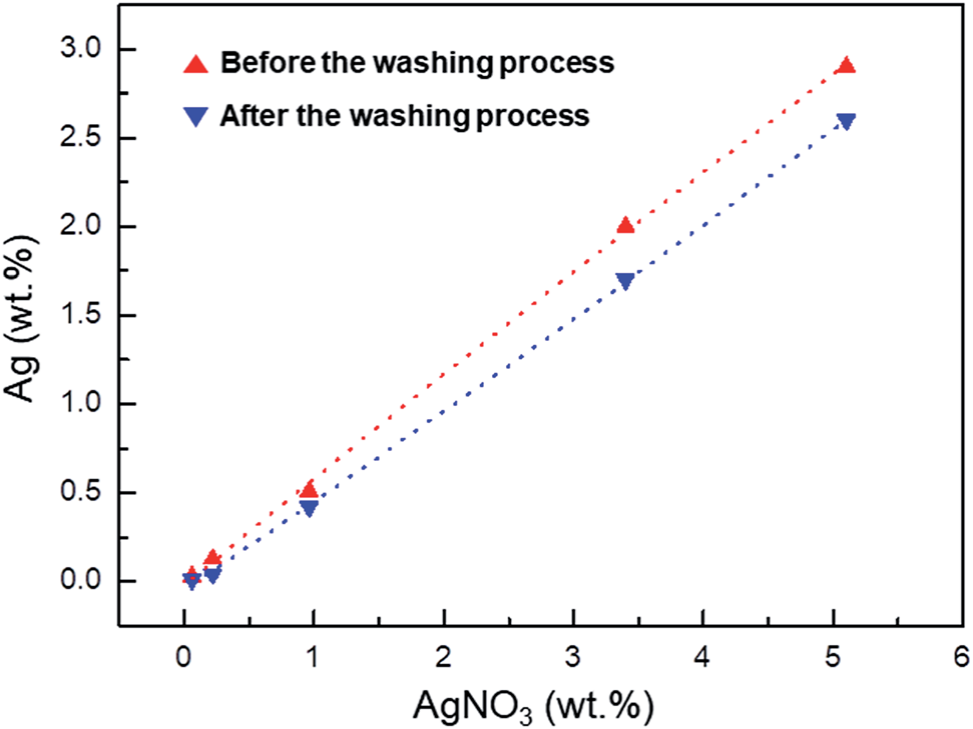
Inductively coupled plasma (ICP) data of Ag atoms in films with 0.06, 0.22, 0.96, 3.40, and 9.10 wt% AgNO_3_ affected by the washing process.

As shown by the ICP data in Fig. 3, the washing process removes unreacted Ag^+^, reducing the Ag^+^ concentration in the Ag^+^/AgNP/PVDF composite film. The conversion rate of Ag^+^ to AgNPs is calculated by the Ag wt% value present in the sample before and after washing. These values are 37, 31.5, 82.4, 85, and 89.7% for 0.06, 0.22, 0.96, 3.40, and 5.10 wt% AgNO_3_, respectively. As the concentration of AgNO_3_ in the sample is increased, the amount of Ag^+^ that can be converted into AgNPs is also increased, thus increasing the Ag^+^–AgNP conversion.

**Fig. 4:**
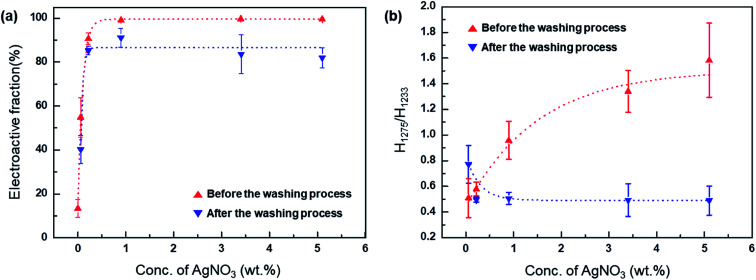
(a) Electroactive fractions of films with 0.06, 0.22, 0.96, 3.40, and 9.10 wt% AgNO_3_, as measured before and after washing process. (b) Ratio of β- to γ-phases (*H*_1275_/*H*_1233_) as a function of AgNO_3_ contents in AgNP/PVDF composites films.

The fractions of the electroactive phase (β + γ) in the PVDF composite film can be quantitatively calculated using the following equation:^47^2
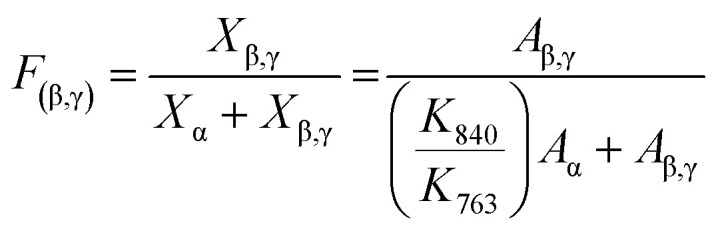
where *X* is the crystalline mass fraction, *A* is the absorbance of specific peaks (α shows an absorbance peak at 764 cm^−1^ and β + γ shows a peak at 840 cm^−1^), and *K*_840_ and *K*_763_ are the absorption coefficients at the respective wavenumbers with values of 7.7 × 10^4^ and 6.1 × 10^4^ cm^2^ mol^−1^, respectively. The fractions of (β + γ) in the PVDF composite films according to eqn (2) are plotted in Fig. 4(a). These results indicate that for the Ag^+^/AgNP/PVDF composite films, the highest fraction of the electroactive phase is increased from 55% to 90% as the amount of AgNO_3_ is increased from 0.06 wt% to 0.22 wt%. As the amount of AgNO_3_ is increased from 0.22 to 5.10 wt%, the fraction of the electroactive phase remains almost constant, reaching a maximum value of 99%. In contrast, for the AgNP/PVDF film, the fraction of the electroactive phase of PVDF is increased more significantly with increasing AgNO_3_ than that for Ag^+^/AgNP/PVDF. In addition, the amount of the electroactive phase in PVDF is gradually increased to 86% until the amount of AgNO_3_ reaches 0.96 wt%. However, as AgNO_3_ is increased from 0.96 wt% to 5.10 wt%, the value of the electroactive phase is gradually decreased to 80%. These results confirm that Ag^+^ and AgNPs are highly effective in inducing electroactive phase formation in PVDF.

The fractions of the electroactive phase were calculated using the absorbance of the peak at 840 cm^−1^. However, this peak reflects both β- and γ-phases. To confirm the effect of Ag^+^ on β phase induction, quantitative values of β- and γ-phases were needed. The quantification of individual β- and γ-phases was performed using the absorbance of the two peaks (1275 and 1234 cm^−1^) and the following equation:3
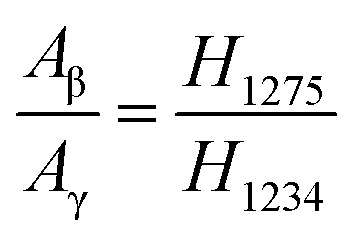
where *A*_β_ and *A*_γ_ are the absorbance of peaks at 1275 and 1234 cm^−1^, respectively. For the Ag^+^/AgNP/PVDF composite film, *A*_γ_ > *A*_β_ from 0.06 wt% to 0.96 wt% of AgNO_3_, as shown in Fig. 4(b). However, as the amount of AgNO_3_ is increased from 0.96 to 5.10 wt%, *A*_β_ > *A*_γ_ because Ag^+^ induce β-phase formation. The highest ratio of the β-phase in the total polar phase is 61%. Meanwhile, for the AgNP/PVDF composite film, *A*_γ_ > *A*_β_ for all AgNO_3_ weight fractions. Additionally, as the concentration of AgNO_3_ is increased, *A*_γ_ is increased but *A*_β_ is not. The ratio of the γ-phase in the total polar phase is increased to 67%, 28% higher than that before the washing process. These results indicate that, in AgNP/PVDF composite films, the ratio of β- and γ-phases in the electroactive phase can be controlled using the washing process described here; Ag^+^ supports β-phase while AgNPs induce γ-phase.

## Experimental

### Materials

The PVDF polymer was Solef® 6008 (Solvay Solexis) and the solvent was *N*,*N*-dimethylformamide (DMF, Sigma-Aldrich, USA). AgNO_3_ (Sigma-Aldrich, USA) was used as an Ag^+^/AgNP precursor. Hydrazine monohydrate (NH_2_NH_2_·H_2_O, Junsei Chemical, Japan), in the same molar ratio as AgNO_3_, was added as a reducing agent to the DMF/PVDF/AgNO_3_ system to reduce Ag^+^ to AgNPs.

### Methods

#### Preparation of Ag^+^/AgNP/PVDF solution

PVDF was dissolved in DMF for 4 h at 60 °C to prepare a 10 wt% solution, and cooled to room temperature to which AgNO_3_ solution at different concentrations were mixed. To determine the effect of AgNO_3_ concentration, AgNO_3_ solutions of concentrations 0.06, 0.22, 0.96, 3.40, and 5.10 wt% were formed and stirred at a constant speed for 10 min. Then, hydrazine was added to the solution in the same molar ratio as AgNO_3_ and reacted for 3 h to prepare an AgNP/PVDF composite.

Half of each reaction solution was dried in a vacuum oven at 80 °C for 24 h; this portion was used to form Ag^+^/AgNP/PVDF films. The remaining half was subjected to a washing process to remove Ag^+^ and form AgNP/PVDF composite films. The solution was washed three times in triple distilled water. In this process, the reaction solution was precipitated in water, stirred for 1 h, filtered, and dried in a vacuum oven at 80 °C for 24 h followed by re-dissolution in DMF. The washing–precipitation–re-dissolution procedure was repeated five times for each reaction solution. Finally, the unwashed and washed samples were dissolved in DMF to prepare 10 wt% casting solutions of equal concentrations.

#### Preparation of AgNO_3_/AgNP/PVDF and AgNP/PVDF composite films

A section of polyethylene terephthalate (PET) film (7 × 21 cm^2^) was heated to 80 °C and fixed to prevent movement during casting. The prepared unwashed casting solution of AgNO_3_/AgNP/PVDF (2.0 mL) was coated onto the PET film and dried for 5 min at 80 °C before 6 h drying in a convection oven at 80 °C to remove the remaining solvent. This process was repeated for each concentration of washed and unwashed AgNPs/PVDF casting solution to obtain 11 total film samples.

### Characterization

The crystalline phases of PVDF were identified by Fourier-transform infrared spectroscopy (FT-IR, Bruker ALPHA-T & ALPHA-P). The film morphologies were investigated by field-emission scanning electron microscopy (FE-SEM, TESCAN Mira 3 LMU FEG) and energy dispersive spectroscopy (EDS, Bruker Quantax 200). Inductively coupled plasma-atomic emission spectroscopy (ICP-AES, Thermo Fisher Scientific iCAP 6500Duo) was conducted to determine the relative amount of Ag^+^ to NP conversions before and after washing. A differential scanning calorimeter (DSC, Q1000 V9.9 Build 303) was calibrated with in before use and calorimetry was performed in N_2_ atmosphere. The films were heated at 5°C min^−1^ from 0 to 220 °C.

## Conclusion

Polar crystal phase formation was induced in PVDF through the preparation of PVDF composite films with Ag^+^ and AgNPs. In addition, the concentrations of β- and γ-phases were controlled using a novel washing process. The polarity of the Ag^+^/AgNP/PVDF composite film before washing reached 98%, and the ratio of the β phase was 61%. The polarity of the composite film after washing was 85%, lower than that before washing. However, of the total polar phase of PVDF, the ratio of the β phase was decreased and the ratio of the γ-phase was increased to 67%, demonstrating the ability of Ag^+^ and AgNPs to induce β- and γ-phase formation, respectively. These results indicated that the polar phase and, thus, polarity of PVDF can be easily adjusted, thereby promoting various applications such as outdoor energy harvesting, self-powered wearable devices, and portable sensors.

## Conflicts of interest

The authors declare no competing financial interest.

## Supplementary Material

RA-009-C9RA08763J-s001
