# Supplementary material for: Preparation and electroactive phase adjustment of Ag-doped poly(vinylidene fluoride) (PVDF) films
Source: RSC Adv. 2019 Dec 4;9(69):40286–91. doi: 10.1039/c9ra08763j (PMC9076169; doi:10.1039/c9ra08763j)
Supplement: RA-009-C9RA08763J-s001 [file RA-009-C9RA08763J-s001.pdf]

Electronic Supplementary Material (ESI) for RSC Advances.
This journal is © The Royal Society of Chemistry 2019

Supporting Information

Preparation and electroactive phase adjustment of Ag-doped poly (vinylidene fluoride) (PVDF) films

Seung-Hyun Kim[a,b,], So-Jeong Park[a], Chang-Yeol Cho[a], Hong Suk Kang[a], Eun-Ho Sohn[a], In Jun Park[a], Jong-Wook Ha[a] and Sang Goo Lee[a,*]

[a] Interface Materials and Chemical Engineering Research Center, Korea Research Institute of Chemical Technology, Daejeon, 34114, Republic of Korea

[b] School of Chemical Engineering, Sungkyunkwan University, Suwon 16419 Republic of Korea

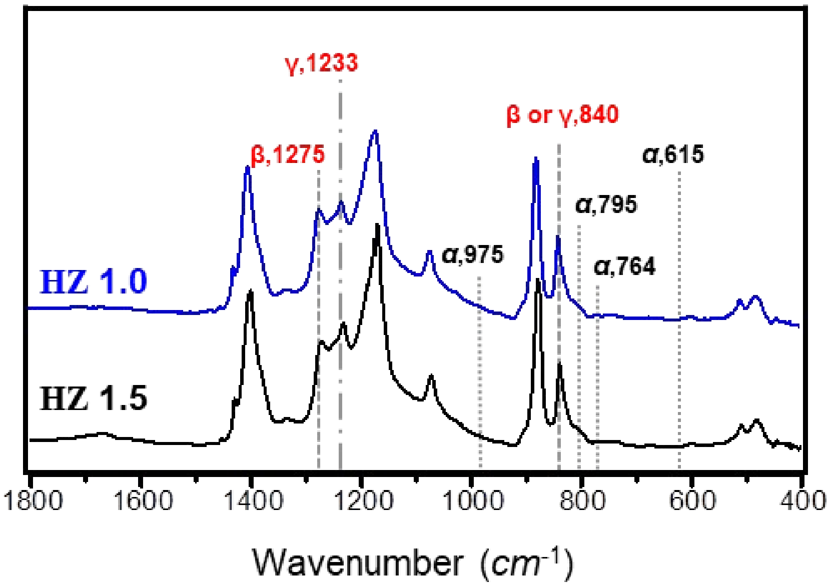

Figure S1. FT-IR spectra for Ag^+/AgNP/PVDF composite film according to the amount of hydrazine.

Table S1. The melting enthalpy (ΔH_f) and total crystallinity (X_c) of PVDF films with 0.06, 0.22, 0.96, 3.40, and 5.10 wt.% $AgNO_3$, as measured before and after washing process.

	Before washing		After washing	
	Melting Enthalpy (J/g)	Crystallinity (%)	Melting Enthalpy (J/g)	Crystallinity (%)
0	43.63	41.67	43.63	41.67
0.06	43.84	41.87	44.25	42.26
0.22	44.95	42.93	44.50	42.50
0.96	44.82	42.81	44.64	42.64
3.4	44.05	42.07	44.17	42.19
5.1	45.37	43.33	44.72	42.71

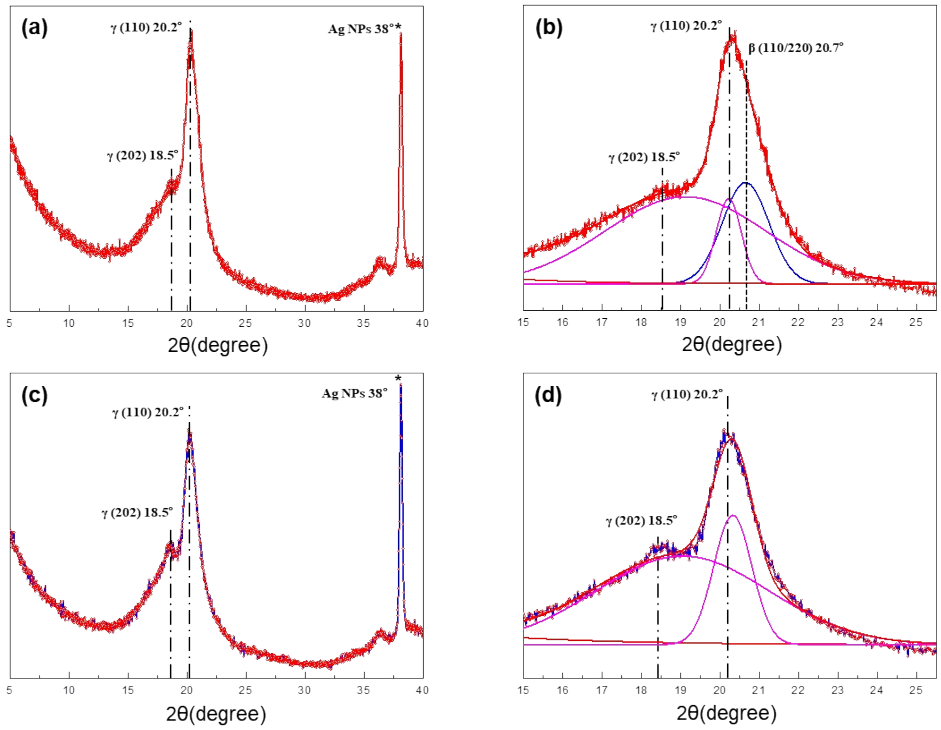

Figure S2. XRD patterns and their curve deconvolution of (a)(b) PVDF-Ag 3.40 wt.% before washing process, (c)(d) PVDF-Ag 3.40 wt.% after washing process. The dotted points are experimental data, and the solid lines correspond to the best curve fit. the peaks marked with * correspond to Ag nanoparticles.
